# Mammary epithelial cell exfoliation increases as milk yield declines, lactation progresses, and parity increases

**DOI:** 10.3168/jdsc.2023-0534

**Published:** 2024-04-20

**Authors:** H.H. Webster, A.J. Lengi, B.A. Corl

**Affiliations:** Department of Dairy Science, Virginia Tech, Blacksburg, VA 24061

## Abstract

Milk yield (MY) naturally declines after peak lactation. One approach to improving production is to increase lactation persistency, or the length of time a cow maintains lactation. To do so, cows must maintain functional secretory mammary epithelial cells (MEC), which synthesize milk. Some viable MEC are exfoliated from the udder into milk. The objective of this experiment was to identify the magnitude and direction of association between exfoliated MEC and MY, days in milk (DIM), and parity. Our hypothesis was that MY and the quantity of exfoliated MEC were negatively correlated. Therefore, milk from low-yielding, late-lactation, and primiparous cows would contain more MEC. Thirty-six multiparous and 12 primiparous Holstein cows were sampled once during lactation. Flow cytometry was used to identify hematopoietic cells and MEC in milk using an antibody specific for cluster of differentiation-45 (CD45) and an antibody against butyrophilin 1A1 (BTN), respectively. Nucleated cells were stained with Hoechst dye, and propidium iodide was used to identify dead cells. For statistical analysis, we analyzed the effect of parity, MY, and DIM, and DIM^2^ on the number and concentration of both total and live exfoliated MEC. Primiparous cows exfoliated fewer MEC and had a lower concentration of MEC in milk than multiparous cows regardless of lactation stage. This could reflect continued mammary growth in first lactation. Cows shed an average of 9.80 × 10^7^ ± 6.90^7^ MEC (LSM ± SEM) with a concentration of 4,582 ± 3,574 cells/mL in one milking. Milk yield was inversely related to concentration of both total and live MEC in milk. Similarly, cows in late lactation, which is associated with MY decline, exfoliated a greater number of MEC into milk. Primiparous cows had a lower MY and shed fewer total and live MEC in this study. The relationship of MY and DIM with MEC losses in milk may also contribute to declining MEC number in the mammary gland found in postpeak lactation. Understanding MEC exfoliation regulation could help develop husbandry practices that improve secretory cell retention in the mammary gland and increase lactation persistency.

## Highlights

• Anti-butyrophilin antibodies were used to evaluate exfoliated mammary cells in milk.
• First-lactation cows exfoliated fewer mammary cells into milk than older cows.
• Lower milk yield was associated with a greater concentration of cells in milk.
• Late-lactation cows exfoliated more cells into milk than early-lactation cows.

## Graphical Abstract

**Figure:**
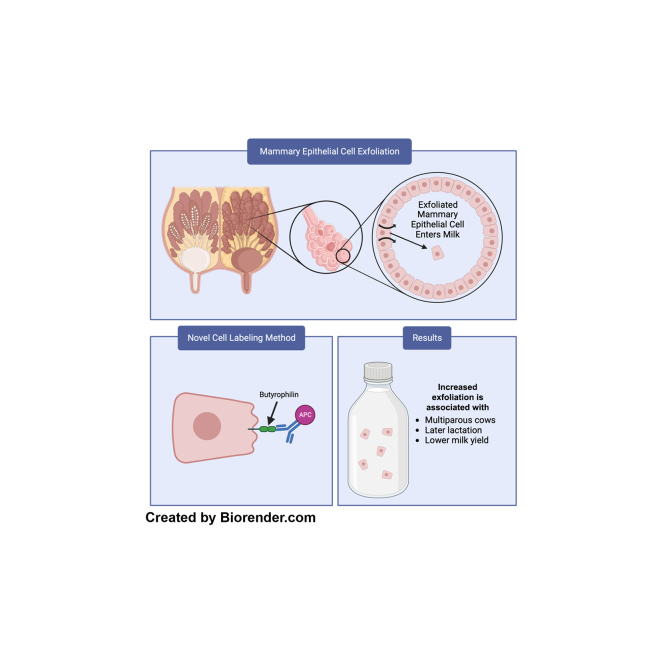
**Summary:** Mammary epithelial cells are responsible for producing milk in the mammary gland. A small fraction of these cells is exfoliated into milk by the mammary gland while still alive. This study aimed to determine an association of live and total mammary epithelial cell number and concentration in milk with milk yield, days in milk, and parity using butyrophilin as a novel marker to label secretory cells. Cows that produced less milk and those in advanced lactation stages had higher concentrations of mammary epithelial cells in milk. Compared with multiparous cows, primiparous cows had fewer mammary epithelial cells in milk.

Mammary epithelial cells (**MEC**) produce milk in the mammary gland. Cell turnover, via proliferation and apoptosis, in the mammary gland is a natural tissue process throughout lactation. The decline in MEC number is generally associated with apoptosis, but other processes such as efferocytosis, autophagy, and exfoliation could also result in mammary gland cell losses (Boutinaud and Jammes, 2002). Exfoliation is defined as the shedding of MEC into milk (Herve et al., 2016). Previous literature has also described this type of epithelial cell loss as extrusion, or the act of an epithelial cell being detached from the basement membrane by the forces of surrounding cells (Kuipers et al., 2014). While some MEC in milk are dead or dying, others are still alive, which seems counterintuitive to the maintenance of lactation (Boutinaud et al., 2013a). Additionally, exfoliation of live MEC notably occurs during milking (Herve et al., 2017). Exfoliation can occur during milking due to oxytocin release at milk letdown, which causes the contraction of myoepithelial cells (Herve et al., 2017). Cell crowding has also been shown to lead to exfoliation of live epithelial cells (Eisenhoffer et al., 2012). This type of cell loss has proven to be useful as a noninvasive alternative to mammary biopsies because live cells in milk reflect the mammary gland without the disturbance of surgical biopsy (Boutinaud et al., 2015; Sharma et al., 2019).

Identifying and quantifying MEC in the heterogeneous somatic cells of milk requires selective techniques. Generally, the majority of milk somatic cells are involved in immunity. Anti-cluster of differentiation-45 (CD45) antibodies can be used to label immune cells in milk (Boutinaud and Jammes, 2002). This study used a novel MEC labeling method using anti-butyrophilin antibodies. Butyrophilin (BTN) is a membrane-bound glycoprotein specific to MEC that is concentrated on the apical side of the cell membrane due to its role in milk fat secretion (Mather and Jack, 1993; Keenan et al., 2002). Using this antibody fulfilled several requirements for our analysis. As it is a cell surface protein of MEC, we were able to preserve the integrity of the cell membrane and use propidium iodide (**PI**) to identify living cells within milk. By using a protein that is involved in milk fat secretion, we are able to quantify secretory epithelial cells, excluding nonsecretory MEC found in milk that originate from ducts.

Mammary epithelial cell shedding into milk may increase with decreased milk yield (**MY**). Many factors cause MY decline such as lactation progression, feed restriction, reducing milking frequency, increasing milking interval, inhibition of prolactin, pregnancy, incomplete milking, and disease (Herve et al., 2016). Additionally, low MEC exfoliation was linked to increased lactation persistency in ovariectomized cows (Boutinaud et al., 2013b). Researchers found that on average 3.90 × 10^6^ MEC are lost each day through exfoliation (Herve et al., 2016). The objective of this experiment was to enhance understanding of losses of MEC from the udder into milk, especially while cells were still viable. We aimed to achieve this by associating MY with the number and concentration of live and total MEC in milk from cows across lactation stages and parities. We hypothesized that the number of MEC exfoliated into milk would be negatively associated with MY. Therefore, low-production cows would have more exfoliated cells in milk, and primiparous cows would shed more cells because they produce less milk that multiparous cows. This hypothesis is predicated on specific regulation of exfoliation into milk. This relationship could indicate that MEC exfoliation is another mechanism to control MEC number and MY. This could be responsive to physiological or environmental conditions.

All animal procedures were approved by the Virginia Tech Institutional Animal Care and Use Committee (#19–165). Cows were housed at Virginia Tech's Kentland Farm in freestalls and milked twice daily at 0100 and 1300 h. Forty-eight Holstein cows were sampled once during lactation. To capture a wide range of lactation stages, 4 cows were selected the day before sampling to include an early- (25–59 DIM), peak- (60–99 DIM), mid- (100–249 DIM), and late-lactation (≥250 DIM) cow. Only 4 cows were sampled on each sampling day due to constraints related to sample processing for analysis. One primiparous cow was included at each sampling. Of all sampled cows, 48% were gestating at the time of sampling including 76% of cows 100 to 249 DIM and 86% of cows >250 DIM. Selected cows were free of clinical mastitis and had SCC less than 264,000 cells/mL at the previous monthly DHIA test. A single ∼3.8 L milk sample was taken at the morning milking between 0100 and 0400 h from each cow, and an additional 50-mL milk sample preserved with bronopol was collected for composition analysis. Sample size was determined by power analysis to achieve a power of 0.8.

Procedures for preparing milk somatic cells for flow cytometry analysis are described in detail by Lengi et al. (2021). Cells were counted using a hemocytometer, and adjusted to 2.0 × 10^6^ cells per sample. Primary antibodies, anti-BTN-APC (Novus Biologicals MAB8467APC, 7.0 ng/µL) and anti-CD45-PE (Novus Biologicals NB500–492PE, 26 ng/µL), were added and incubated for 1 h at room temperature in the dark. After washing, Hoechst 33342 (Invitrogen, H3570, 10 µg/mL) and PI (BD Biosciences 556463, 5 µg/mL) stains were added, and the cells were incubated for 1 h at room temperature in the dark. Cells were washed again and analyzed by flow cytometry (BD FACSARIA Fusion Flow Cytometer) to measure the percent of MEC (BTN+CD45-) and the percent of immune cells (CD45+BTN-). Somatic cell count was determined by Lancaster DHIA (Lancaster, PA) using a Foss Fossomatic FC (Foss North America).

Statistical analysis was performed using the GLM procedure of SAS version 9.4 (SAS Institute Inc.) to create the multilinear regression model. The dependent variables were the number (cells) or concentration (cells/mL) of both total and live exfoliated MEC for a single milking. Independent variables were MY, DIM, DIM^2^, and parity. Parity was a binary variable of either multiparous or primiparous cows. In the regression equation, primiparous cows were coded as 1 and multiparous cows were coded as 0. Milk yield, DIM, and DIM^2^ were continuous variables. Interactions between all fixed effects were tested and were not significant, so they were not included in the final model. DIM^2^ was included in the model to better fit the curve of lactation. Cell number and concentrations were log-transformed to achieve normally distributed data. Significance was declared at *P* < 0.05 and a tendency was declared at *P* < 0.10. PROC CORR in SAS was used to analyze the correlations between total and live BTN+ exfoliated cells in milk as well as the total and live concentration of BTN+ shed cells in milk with MY and DIM. Concentrations of MEC were calculated by multiplying the percentage of live and total BTN+CD45− cells, determined from flow cytometry, by SCC. Number of live and total BTN+ cells were calculated by multiplying concentrations of MEC in milk by milk volume. The number of exfoliated MEC was calculated for one milking and does not correspond to an exfoliation rate.

As lactation progresses, MY declines naturally due to allocation of energy to other bodily functions such as fetal development. Cows were sampled across lactation stages and parities with 2.6 fold variation in MY. Cows ranged from 25 to 307 DIM with a mean of 143 ± 82 DIM. The mean lactation number was 2.6 and ranged from 1 to 6 lactations. The mean MY for a single milking was 23 ± 5.5 kg (SD; range 14–37 kg). Descriptive statistics of MEC exfoliation from all cows are found in Table 1. Milk from primiparous cows contained an average of 4.66 × 10^7^ ± 2.62 × 10^7^ total MEC of which 8.98 × 10^6^ ± 1.30 × 10^7^ cells were live. Primiparous cows had a concentration of 2,543 ± 1,295 MEC (cells/mL) of which 489 ± 624 cells/mL were live. Milk from multiparous cows contained 1.15 × 10^8^ ± 7.05 × 10^7^ total MEC of which 2.78 × 10^7^ ± 3.13 × 10^7^ were live. Multiparous cows had a total concentration of 5,261 ± 3,838 MEC (cells/mL) of which 1,353 ± 1,829 cells/mL were live. Cows in the experiment had a large variation in live exfoliated MEC. The mean live MEC made up 23% of the total exfoliated MEC in milk with a mean of 2.4 × 10^7^ ± 2.9 × 10^7^. Similarly, live MEC concentration made up 23% of total MEC concentration in milk. Multiparous cows alone had a correlation tendency between MY and number of total (r = −0.29) and live (r = −0.43) shed MEC (*P* < 0.1; Figure 1). For all cows, MY was significantly correlated with both the concentration of total (r = −0.3) and live (r = −0.3) MEC (*P* < 0.05; Figure 1). Cows with higher MY also had more hematopoietic cell yield in milk; MY and CD45+ cells were positively related (*P* = 0.01; not shown).

**Table 1:** Descriptive statistics of total and live milk MEC yields as well as the total and live MEC concentration in milk^1^

Item	Mean	SD	Minimum	Maximum	*P*-value			
					MY	DIM	DIM^2^	Parity
Total MEC yield (cells)	9.8 × 10^7^	6.9 × 10^7^	1.1 × 10^7^	2.6 × 10^7^	ns	0.02	0.02	0.002
Live MEC yield (cells)	2.3 × 10^7^	2.9 × 10^7^	1.6 × 10^6^	1.2 × 10^8^	ns	0.09	ns	0.007
Total MEC concentration (cells/mL)	4,582	3,574	451	17,600	0.002	0.02	0.03	0.002
Live MEC concentration (cells/mL)	1,137	1,651	45	8,160	0.04	ns	ns	0.007

1 Significance was determined from regression analysis of log-transformed values with milk yield, DIM, DIM^2^, and parity variables analyzed in the model. ns = not significant.

**Figure 1:**
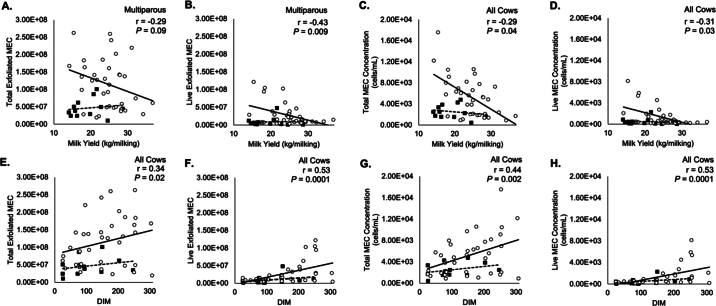
Panels A–D represent correlations between MY and shed MEC, specifically total (A) and live (B) shed MEC as well as total (C) and live (D) concentrations (cells/mL) of MEC in milk from a single milking. Panels E–H represent correlations between DIM and shed MEC, specifically total (E) and live (F) shed MEC as well as total (G) and live (H) concentrations (cells/mL) of MEC in milk. Multiparous cows are indicated by open circles and a solid line. Primiparous cows are indicated by solid squares and a dashed line. Correlation coefficients were determined through Pearson correlation analyses. The *P*-values and correlation coefficients represent combined values from multiparous and primiparous cows excluding panels indicated by “multiparous.” Panels that present multiparous *P*-values and correlation coefficients indicated that only multiparous cows had a significant relationship. Combined correlations for all cows in these panels were not significant. There were no significant correlations between exfoliated MEC and MY or DIM in primiparous cows alone in all panels.

Milk yield declines after peak lactation. One goal of this study was to determine if DIM was associated with MEC exfoliation into milk. There was a significant correlation between the total (r = 0.34) and live number of MEC (r = 0.53) as well as the concentration of total (r = 0.44) and live (r = 0.53) MEC in milk with DIM (*P* < 0.05; Figure 1). Mammary epithelial cell exfoliation increases as lactation progresses. Milk yield was significantly correlated with the concentration of both total (r = −0.3) and live (r = −0.3) exfoliated MEC (*P* < 0.05). Pearson correlations showed that low production multiparous cows had even greater concentrations of total (r = −0.56) and live (r = −0.51) MEC in milk (*P* < 0.05; Figure 1). Cows with higher MY also had more hematopoietic cell yield in milk; MY and CD45+ cells were positively related (*P* = 0.01; not shown). A correlation between the ratio of live to total shed MEC and DIM showed that later-lactation cows shed a higher proportion of live MEC into milk compared with early-lactation cows. It is interesting to note that not all later-lactation cows showed cell loss into milk, indicating a high variability between cows even of similar lactation stage. There was no correlation between the ratio of live to total shed MEC and MY. There was also no correlation between the ratio of live to total MEC and fat or protein yields (not shown). Correlations showed no significant relationship between either MY or DIM with exfoliated MEC in primiparous animals alone (Figure 1).

Multilinear regression analysis corresponded with correlation results, indicating that on average, MEC shedding increases with DIM and decreases with MY (Figure 2). However, total MEC shedding was greatest in mid lactation, corresponding to greater MY (Figure 2E and 2G). This was not true of live cell shedding, which was greatest at the end of lactation (Figure 2F and 2H). Milk yield did not affect exfoliation of total or live MEC. However, increasing MY did correspond to a lower concentration of live and total MEC shedding. Within each parity group, late-lactation cows (300 DIM) shed the least MEC and had the lowest concentration of MEC (Figure 2A and 2C), but they had the most live MEC and highest concentration of live MEC in milk (Figure 2B and 2D). An increase in live MEC exfoliation in late lactation could be due to the allocation of nutrients to other physiological processes such as late-gestation fetal growth and the progression toward involution.

**Figure 2:**
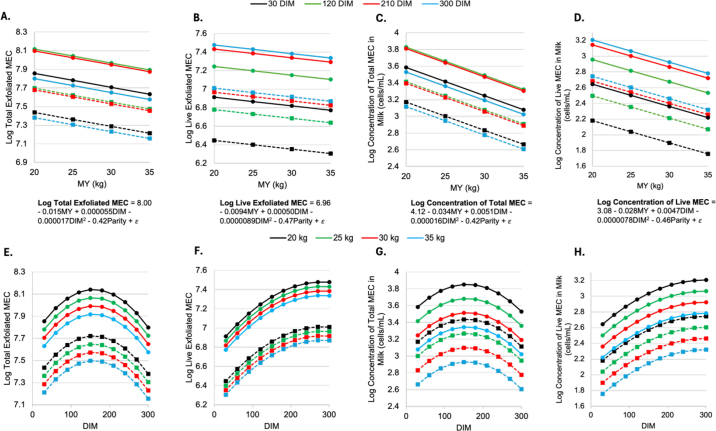
Panels A–D represent the fitted values of a linear regression model between MY and log-transformed shed MEC. Total (A) and live (B) shed MEC and total (C) and live (D) concentrations of MEC in milk (cells/mL) at various DIM. Panels E–H present fitted values of a linear regression model between DIM and shed MEC, specifically total (E) and live (F) shed MEC as well as total (G) and live (H) log-transformed concentrations of MEC in milk (cells/mL) at various milk yields. Multiparous cows are indicated by solid lines and circles. Primiparous cows are indicated by dashed lines and squares.

Parity significantly affected all dependent variables in the model: the log of total and live BTN+ shed cells and their concentration in milk. On average, primiparous cows shed fewer cells into milk and had lower concentrations of cells in milk than multiparous cows (*P* < 0.05; Figure 2). The least squares means number of shed MEC from primiparous cows was not different from zero. The modeled data showed that low-production primiparous cows had a greater concentration of MEC than high-production multiparous cows (Figure 2G). Later-lactation (210+ DIM) primiparous cows shed more live cells (Figure 2B) and had a greater concentration of live cells in milk (Figure 2D) than early-lactation multiparous cows. We hypothesize that primiparous animals shed an insignificant number of MEC because of their ongoing mammary development throughout their first lactation. Mammary growth requires greater cell proliferation than cell loss. Mammary epithelial cell exfoliation would be counterintuitive to mammary growth during this stage of development.

This experiment demonstrated that lower MY is associated with a greater concentration of shed MEC similar to the findings of Herve et al. (2016) in which MY and exfoliation rate (cells/d) were associated. We considered that the relationship between MY and MEC concentration in milk could be influenced by milk volume dilution. However, we determined that concentration may provide a more meaningful relationship than total MEC yield in milk. Milk yield is used to determine the total number of somatic cells in a milk sample. Correlating MY with MEC concentration instead of MEC number may be a better representation of their relationship because MEC number is calculated using MY.

The concept of declining MY increasing the number or concentration of exfoliated MEC into milk has been demonstrated in many studies by Boutinaud and colleagues. Though not the main objective of their research, these studies altered MY through feed restriction (Boutinaud et al., 2008; Herve et al., 2019), reduced milking frequency (**MF**) (Boutinaud et al., 2013a), hormonal manipulation (Boutinaud et al., 2012; Lollivier et al., 2015; Herve et al., 2018), and ovariectomies (Boutinaud et al., 2013a). In a feed restriction study, Herve et al. (2019) found that a 20% feed restriction caused a 3 kg/d decline in MY and a 65% greater rate of MEC exfoliation into milk. The proportion of viable cells also tended to be greater in feed-restricted cows compared with cows fed ad libitum. Like our study, there was an inclination that a reduction in MY leads to greater MEC shedding. Like feed restriction, reduced MF also decreased MY and increased milk MEC exfoliation in goats milked once-daily (1×) compared with twice daily (2×) (Ben Chedly et al., 2013). After 28 d of 1× milking, both milk MEC concentration (cells/mL) and rate of shedding (cells/d) increased significantly in 1× compared with 2× milking in goats. Similarly, cows milked 1× tended to have a greater rate of MEC loss into milk compared with 2× (Boutinaud et al., 2012); however, these cows were only milked 1× for 7 d. A longer differential milking period may have resulted in a significantly different rate of exfoliation.

In a review of mammary cell exfoliation in ruminants, Herve et al. (2016) reported that 3.90 × 10^7^ MEC were shed into milk per day, representing a significant portion of the cells in the udder. Through their method of milk MEC detection, the researchers observed that 60% to 70% of these cells were viable and fully differentiated. In this study, the mean number of MEC exfoliated during a single milking in a 2× herd was 9.8 × 10^7^ cells, and only 23% of these cells were viable. We also observed that exfoliated MEC number and concentration in milk were highly variable among cows. We hypothesize that this is due to differences in individual mammary function and potentially a response to hormones such as oxytocin, prolactin, and cortisol.

Research has shown that exfoliation of MEC is endocrine regulated. Herve et al. (2017) found that MEC exfoliation increased at milking because of the mechanical forces of myoepithelial cell concentration of the alveoli in response to oxytocin release. They also found that cortisol was negatively correlated with MEC shedding (Herve et al., 2017). In a subsequent study, researchers administered an oxytocin receptor antagonist, atosiban, before milking to obtain milk solely from the gland cistern (Herve et al., 2018). Afterward, cows received oxytocin to stimulate ejection of milk from the alveolar compartment of the mammary gland. They again found that oxytocin caused increased MEC shedding into milk (Herve et al., 2018). Prolactin may also play a role in MEC exfoliation into milk. Lollivier et al. (2015) administered quinagolide to inhibit milking-induced prolactin release. The inhibition of prolactin with quinagolide increased the shedding rate of both live and dead MEC (Lollivier et al., 2015). Prolactin has a cell maintenance function for MEC and, therefore, a lactational maintenance or persistence effect (Lollivier et al., 2015). With reduced prolactin, the increased loss of cells could be the result of exfoliation due to a loss of maintenance of cells and also coincides with declining MY. Therefore, cows that have a strong response to oxytocin or a reduced influence of prolactin on cell maintenance, either through prolactin concentration or receptor activation, may exfoliate more MEC.

In conclusion, we found that lactation progression, MY, and parity affected MEC exfoliation. Cows that produced less milk had greater concentrations of both live and total milk MEC. Late-lactation cows exfoliated more MEC and had a greater concentration of MEC in milk. Finally, milk from multiparous cows contained more and had a greater concentration of total and live MEC than that of primiparous cows likely due to ongoing mammary growth throughout first lactation. Lactation progression is typically associated with an overall decline in MY. We hypothesize that this type of cell loss may be a natural regulator of MY decline over the course of lactation. We cannot say that MEC loss into milk causes a decrease in milk production, only that MEC exfoliation and MY are negatively associated. Future research could include the relationship between number of shed cells and lactation persistency. Cows with a naturally greater lactational persistence, such as those with high genetic merit, may lose fewer secretory cells into milk during peak lactation. Further research could use the BTN cell labeling method alongside annexin and PI, similar to Herve et al. (2017) to determine whether viable cells shed into milk are truly alive or have been targeted for destruction before exfoliation.
